# ASO Author Reflections: Physical Functioning in Patients with Sarcoma After Amputation: Are Current Questionnaires Fit for Purpose?

**DOI:** 10.1245/s10434-026-19474-8

**Published:** 2026-03-13

**Authors:** Tom I. Bootsma, Olga Husson

**Affiliations:** 1 Department of Public Health, Erasmus University Medical Center, Rotterdam, The Netherlands; 2 Department of Medical Oncology, Netherlands Cancer Institute, Antoni van Leeuwenhoek, Amsterdam, The Netherlands; 3 Department of Surgical Oncology, Erasmus University Medical Center, Rotterdam, The Netherlands

Physical functioning (PF) is a central component of health-related quality of life (HRQoL) and is particularly affected in patients with sarcoma who undergo limb amputation. Although commonly used patient-reported outcome measures (PROMs), such as the European Organization for Research and Treatment of Cancer Quality of Life Questionnaire–Core 30 (C30) and Toronto Extremity Salvage Score (TESS), include PF domains, neither instrument has been validated for use in patients with sarcoma after limb amputation.^1,2^ Previous work from our group showed remarkably low PF scores on the C30 among these patients, raising a critical question: how do patients with sarcoma after amputation interpret these items, and do their responses reflect functioning with or without a prosthesis, assistive devices or support from others?^3^

The present study highlights significant challenges in assessing PF in individuals with extremity or pelvic sarcoma after amputation.^4^ Participants described substantial variation in how they approached both the TESS and the C30, often depending on prosthesis or assistive device use, the level of amputation, and the degree of available support. These contextual influences shaped how questions were understood and answered, revealing substantial inconsistency in response patterns. Interpreting these findings through the World Health Organization’s International Classification of Functioning, Disability and Health (ICF) framework underscores that PF cannot be separated from its contextual determinants.^5^ Yet, neither the C30 nor the TESS explicitly incorporates these factors, limiting their accuracy and interpretability for this population.

These observations raise important concerns for both clinical practice and research. If current PF scores reflect different assumptions about how questions should be answered, comparisons across patients, and even within patients over time, may lead to misleading results. A key question for future research is whether explicit, context-sensitive instructions can help patients with sarcoma after amputation to complete PF items more accurately and consistently. A more context-sensitive interpretation of PF will improve the validity and clinical utility of PROMs in sarcoma (survivorship) care.
